# Cost-effective scarless cholecystectomy using a modified endoscopic minimally invasive reduced appliance technique (Emirate)

**DOI:** 10.3389/fsurg.2023.1200973

**Published:** 2023-04-26

**Authors:** Iyad Hassan, Lina Hassan, Mohammad Alsalameh, Hamza Abdelkarim, Wiam Hassan

**Affiliations:** Department of Surgery, Burjeel Hospital, Abu Dhabi, United Arab Emirates

**Keywords:** suprapubic approach, laparoscopic cholecystectomy, innovation & flexibility, case matching, cost-effectiveness, three port cholecyctectomy

## Abstract

**Abstract:**

The current gold-standard surgical treatment for symptomatic gallstone disease is the conventional four-port laparoscopic cholecystectomy (CLC). In recent years, however, celebrities and social media have altered people's attitudes regarding surgery. Consequently, CLC has undergone several changes to reduce scarring and improve patient satisfaction. In this case-matched control study, the cost-effectiveness of a modified endoscopic minimally invasive reduced appliance technique (Emirate) that uses less equipment and three 5 mm reusable ports only at precisely specified anatomical sites was compared to CLC.

**Methods:**

Single-center retrospective matched cohort analysis including 140 consecutive patients treated with Emirate laparoscopic cholecystectomy (“ELC-group”), matched 1:1 by sex, indications for surgery, surgeon expertise, and preop bile duct imaging, with 140 patients receiving CLC in the same period of time (“CLC group”).

**Results:**

We performed a retrospective case-matched review of 140 patients who had Emirate laparoscopic cholecystectomy for gallstones between January 2019 and December 2022. The groups included 108 females and 32 males with an equal ratio of surgical expertise—115 procedures were performed by consultants and 25 by trainees. In each group, 18 patients had preoperative MRCP or ERCP and 20 had acute cholecystitis as indications for surgery. Preoperative characteristics such as age (39 years in the Emirates group and 38.6 years in the CLC group), BMI (29.3 years in the Emirates group and 30 years in the CLC group), stone size, or liver enzymes showed no statistical difference between the two groups. In both groups, the average hospital stay was 1.5 days, and there was no conversion to open surgery, nor was there any bleeding requiring blood transfusion, bile leakage, stone slippage, bile duct injury, or invasive intervention postoperatively. When compared to the CLC group, the ELC group had significantly faster surgery times (*t*-test, *p* = 0.001), lower levels of the bile duct enzyme ALP (*p* = 0.003), and much lower costs (*t*-test, *p* = 0.0001).

**Conclusion:**

The Emirate laparoscopic cholecystectomy method is a safe alternative to the traditional four-port laparoscopic cholecystectomy that is also much faster and less expensive.

## 1. Introduction

The frequency of gallstones is on the rise in Europe and North America, according to ultrasonography studies. Gallstone disease is the second most costly digestive condition in the United States, and about 700,000 cholecystectomies are performed in the US each year (1), while 190,000 patients with gallstones have surgery in Germany (2, 3). **Over the last two decades, the attitudes and expectations of patients have been significantly impacted by celebrities and social media. As a result, the drive for scar reduction and the growing acknowledgment of patient satisfaction have led to the advancement of traditional laparoscopic surgery. As surgeons have gained more experience, both the number of ports and the size of each port have decreased.** Numerous clinical studies have linked variables such as the number and size of ports, the site of the skin incision on the torso, the method of occluding the cystic duct and artery, the method of closing the fascia, the retrieval side of the specimen, and even the exact routing of ports in relation to anatomical landmarks like the falciform ligament to the safety of the treatment and the incidence of complications (4). However, the suprapubic laparoscopic cholecystectomy was initially reported in 1995 in Italy by Degano et al. (5). Today, only a few published articles can be found in the medical literature about this simple technique. This may be related to the widespread interest in alternative minimally invasive methods, such as natural orifice endoscopic translumenal surgery (NOTES) and single-incision laparoscopic cholecystectomy (SILC) (6–8). The mini laparoscopic cholecystectomy (MLC) is another method worth mentioning in this context. Even with the more recent generation of mini instruments, MLC does not offer any clear advantages over conventional laparoscopic cholecystectomy. This is especially true due to the hybrid use of 5 and 10 mm trocars for the camera and the removal of thick-walled gallbladders or large stones, making a pure MLC inapplicable and keeping the technique reserved for only a few selected cases (9). However, considering the global surgical community's strong interest in NOTES and SILC procedures, and significant concerns about the steep learning curve for MLC, safety and cost persist. These factors may help explain why there has been a recent global increase in demand for suprapubic cholecystectomy (10). Because of the low cost of this method without the need for any disposable or specific instruments, and the fact that it maintains the fundamental principles of laparoscopic cholecystectomy, this innovative version makes for a fascinating option that merits more investigation (11).

In this paper, we provide the findings of a retrospective case-matched analysis that evaluated the safety and cost-effectiveness of conventional laparoscopic cholecystectomy (CLC) vs. the endoscopic minimally invasive reduced appliance technique (Emirate).

## 2. Materials and methods

In this study we compared the outcomes of Emirate cholecystectomy (“E-group”) and conventional laparoscopic cholecystectomy (“CLC”) in a tertiary care private hospital in Abu Dhabi, United Arab Emirates, using a retrospective matched cohort analysis of 280 patients treated between January 2019 and December 2022. The patients were matched 1:1 by sex, rationale for surgery, surgeon expertise, and preoperative bile duct imaging. In order to eliminate any possibility of selection bias, the coordinator of the operating room used digital logbooks to conduct a random selection of patients for the control group. Informed consent was obtained from all the patients included in the study. Exclusion criteria were ASA > 3, pregnancy, or refusal to participate in the study. OR-cost, operative time, length of hospital stay, postoperative liver function laboratory test, and conversion to an open or four-port cholecystectomy were evaluated in both groups. A standard case record worksheet was used to gather data on demographic characteristics, pre-operative investigations, and intra- and postoperative parameters.

### 2.1. Equipment

**Figure 1:**
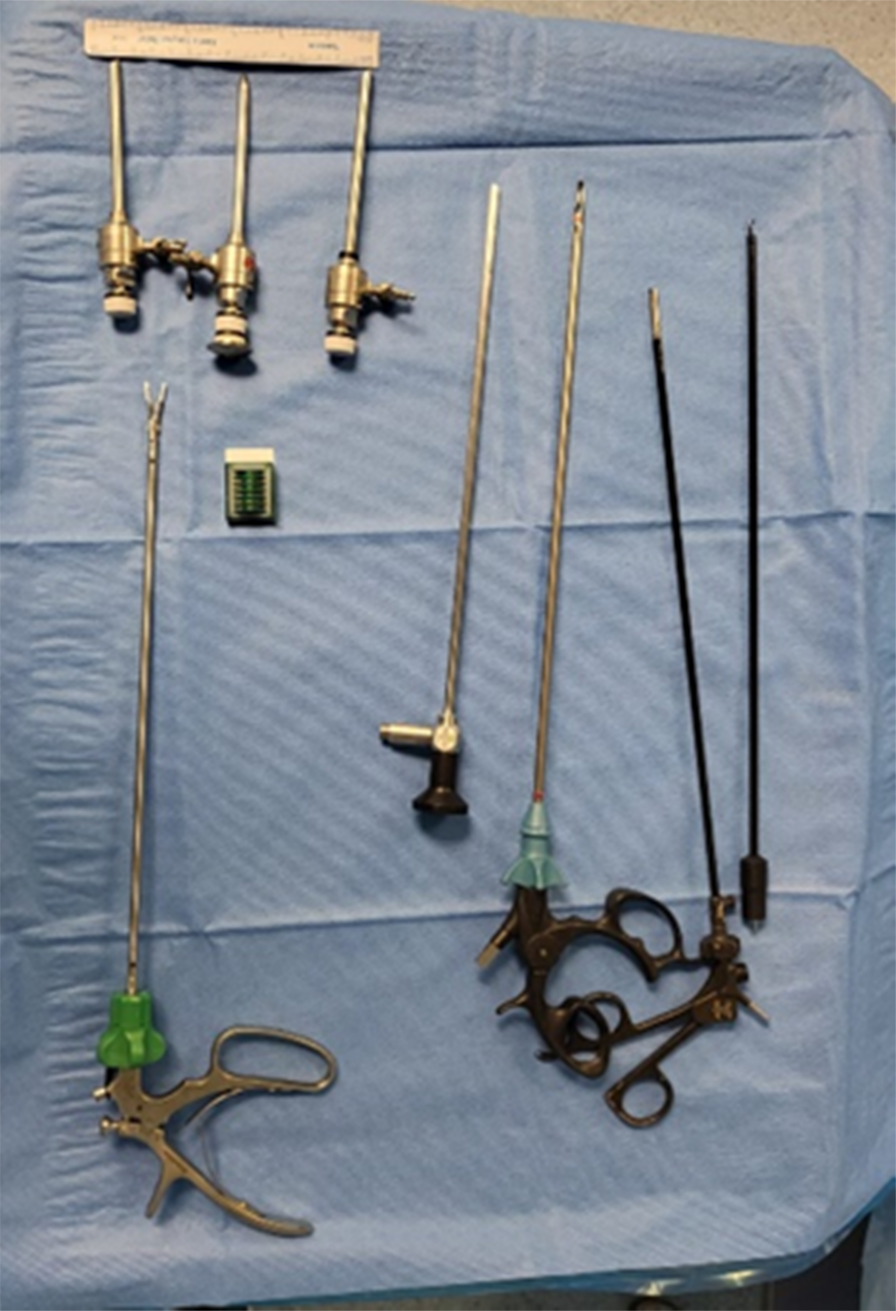
Reduced number of reusable instruments used to perform emirate cholecystectomy (karl storz SE & Co. KG, Tuttlingen, Germany).

Only the following instruments were required for Emirates laparoscopic cholecystectomy: (Figure 1) Veress needle, three 5 mm ports, one grasp instrument, electrocoagulation hook, bipolar Maryland forceps, endoclip applicator, and six polymer-clip cartridges. All the instruments are reusable (Karl Storz SE & Co. KG, Tuttlingen, Germany). A 10 mm trocar and suction device were on hand in case they were needed.

### 2.2. Surgical technique for Emirate cholecystectomy

**Figure 2:**
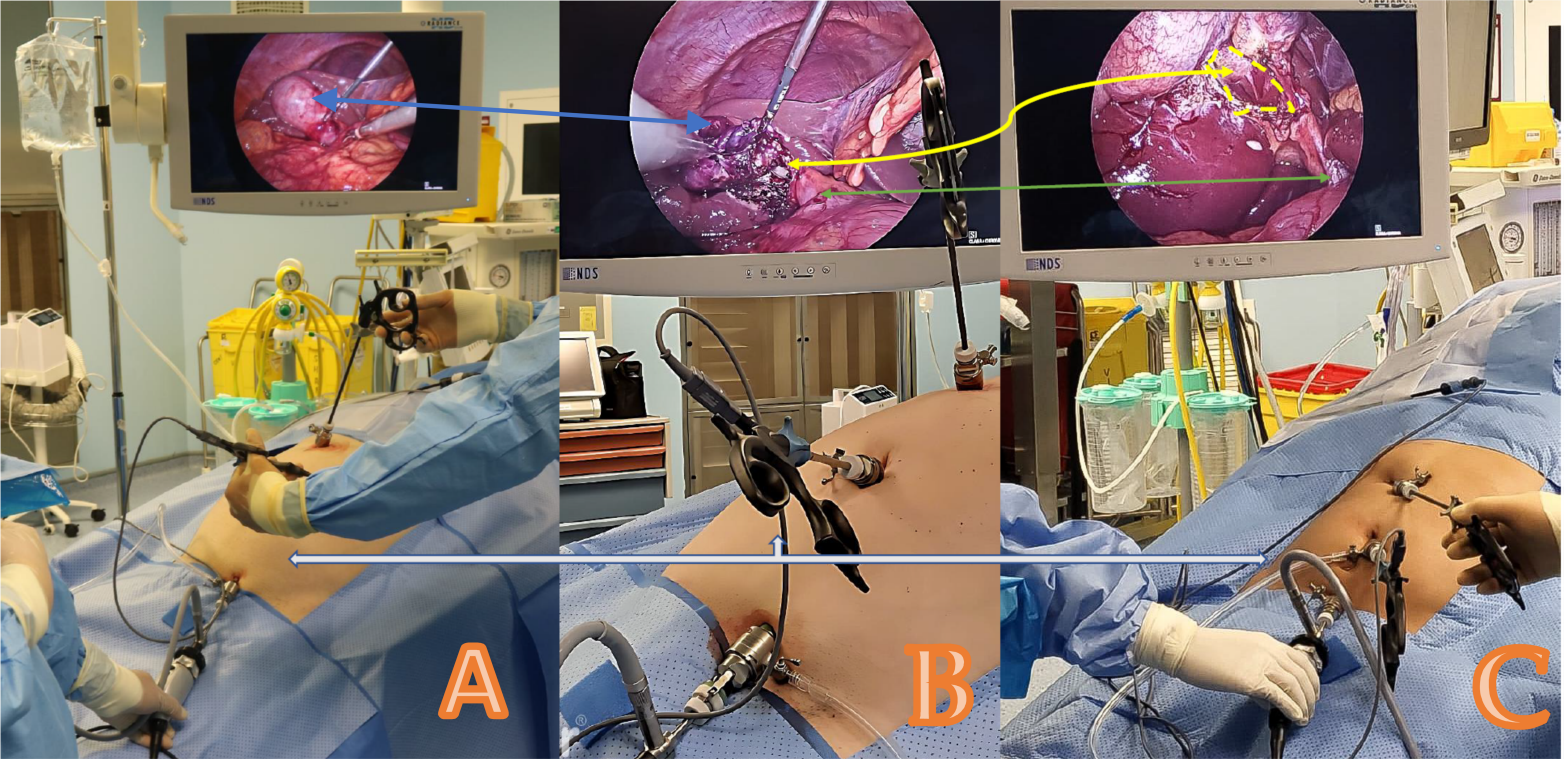
Emirate cholecystectomy showing the standard laparoscopic setup and trocar position (karl storz SE & Co. KG, Tuttlingen, Germany). A case of acute cholecystitis is shown in (**A,B**). Blue arrows indicate the hydropic gallbladder (**A**) and the status after bile aspiration (**B**). Another case of typical stone disorder is shown in (**C**). The green arrow points to the common bile ducts. The white arrow represents the 5 mm trocars with the optic in the suprapubic trocar and the intra-umbilical main working trocar; in (**B**), bipolar forceps are used in that trocar to dissect the calot triangles. The yellow arrow indicates the critical view of safety in both cases of acute and chronic cholecystitis performed *via* the Emirate technique.

**Figure 3:**
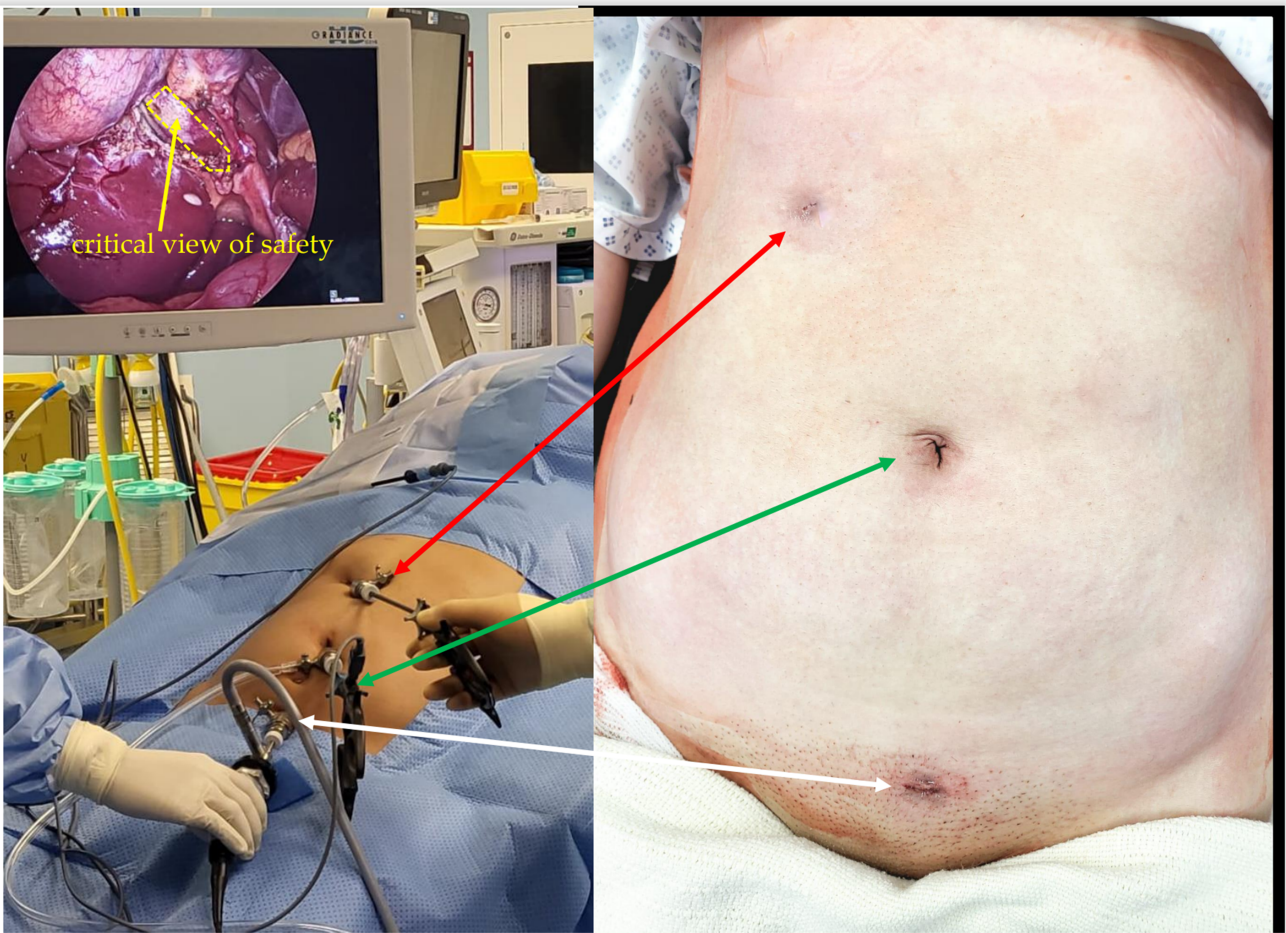
Emirate laparoscopic cholecystectomy showing all 5 mm port positions and final non-visible scars.

Following general anesthetic induction and endotracheal intubation, the patient's positioning and the sequence of the anesthesia provider, instrumenting nurse, and main surgeon are similar to the usual American position for laparoscopic cholecystectomy. The Emirate procedure begins with a 5 mm intraumbilical incision, through which a 5 mm blunt camera port (Karl Storz SE & Co. KG, Tuttlingen, Germany) is inserted. A 5 mm telescope (Karl Storz Image 1 three-chip system, Karl Storz SE & Co. KG, Tuttlingen, Germany) is inserted *via* the intraumbilical camera port, followed by abdominal cavity exploration. Another 5 mm trocar is inserted at the suprapubic hairline; if the pelvis is small, a Trendelenburg posture may be considered for safe trocar placement. Once the precise location and morphology of the gall bladder have been determined, a second 5 mm port is placed below the subcostal edge on the right hypochondrium. The operating surgeon and assistant stand on the patient's left side, while the staff nurse stands on the patient's right. Monitor, insufflation, and light source systems are kept on the foot side of the patient (Figure 2). The 5 mm camera is transferred to the suprapubic port. The bipolar Maryland forceps are inserted *via* the umbilical port and held with the surgeon's right hand to raise the gallbladder fundus above the liver to facilitate optimum grasping of the gallbladder infundibulum through the right subcostal 5 mm port. An alternate peeling approach with bipolar Maryland forceps can now be used to dissect the calot's triangle, forcipes, or monopolar hook from the intraumbilical working trocar. A critical perspective on safety can be seen after the posterior dissection. Bipolar forceps are used to occlude the cystic artery, saving precious time and effort by reducing the need for instrument interchange maneuvers and making the most of the available space for the application of a 5 mm Ham-O-Lock clip. Any bleeding caused by omental adhesion or fat in the gallbladder infundibulum should be stopped as soon as possible with the bipolar forceps. If required, an intraoperative cholangiogram (IOC) is conducted *via* the 5 mm subcostal port using a Fogarty catheter. After dissecting the gallbladder from the gallbladder fossa in the correct plane, the gallbladder specimen is retrieved from the 5 mm suprapubic port after the camera is switched to the umbilical port. The incision and fascia may be expanded by a huge stone larger than 2 cm. After attaining appropriate hemostasis, the suprapubic trocar is removed, and the peritoneum and posterior fasciae are grasped with bipolar forceps. Coagulation is performed to produce tighter and faster scarring of the incision side, as well as to reduce the chance of a trocar hernia. The trocars are then removed under supervision. Rapid Vicryl 4–0 cutting needle sutures are used to close the skin of the port sites (Figure 3).

### 2.3. Conventional laparoscopic cholecystectomy (CLC)

The CLC procedure was carried out as follows. Disposable trocars were used; subcostal and lateral ports of 5 mm, an epigastric port of 10 mm, and an umbilical port of 10 mm (Ethicon Endo-Surgery, Cincinnati, OH, USA) for a 30 ° laparoscope were employed (Figure 2). Furthermore, 5 mm graspers and an electro hook were used (Karl Storz SE & Co. KG, Tuttlingen, Germany). The cystic artery and duct were clipped with a 10 mm applier (Ethicon Endo-Surgery, Cincinnati, OH, USA), and a 10 mm, 30° laparoscope was repositioned into the patient's epigastric port to track the specimen's extraction. The facia was closed with a Vicryl-J-needle strength 1 (Ethicon Endo-suture, Cincinnati, OH, USA).

### 2.4. Statistical analysis

IBM SPSS version 22 was used to carry out the statistical analysis (SPSS Inc., Chicago, IL, USA). The parametric data are reported as a mean with a standard deviation, whereas the non-parametric data are expressed as a median rank with an interquartile range. Student's *t*-tests and Mann–Whitney *U*-tests were used for continuous variables in the univariate analysis, whereas Fischer's exact test was used for categorical variables. A statistically significant *p*-value was defined as one that was less than 0.05.

## 3. Results

### 3.1. Preoperative characteristics

A total of 140 patients underwent laparoscopic cholecystectomy using the novel modified Emirate technique (ELC) between January 2019 and December 2022. These patients were case-matched to 140 patients who underwent laparoscopic cholecystectomy using the standard four-port technique (CLC) over the same time period in our institution. These patients were randomly selected by the Operative Theater Clerk from the operating room logbooks to eliminate selection bias. Both groups were comparable with respect to baseline characteristics such as age, sex composition, BMI, surgeon competence, operative indication, and liver function (Table 1).

**Table 1:** Demographic and pre-operative data for each study group.

Variable	ELC-group (*n* = 140)	CLC-group (*n* = 140)	*p*-value
Age in years	39.05 (11.8)	38.66 (11.5)	0.783*
Female/male	32/108	32/108	1†
Body mass index	29.2 (4.9)	30.1 (5.9)	0.24*
Consultant/trainee	25/115	25/115	1†
Acute/chronic cholecystitis	19/121	19/121	1†
Preop bile duct imaging	18/122	18/122	1†
Previous abdominal surgery	34/106	32/108	0.778†
Preop serum aspartate aminotransferase	48.85 (91.6)	46.99 (111.2)	0.885*
Preop serum alanine transaminase	66.61 (127.1)	57.95 (100.2)	0.559*
Preop serum alkaline phosphatase	88.83 (52.2)	100.51 (70.7)	0.141*

ELC-group = Emirate cholecystectomy; CLC-group = conventional four-port laparoscopic cholecystectomy. Values are presented as the mean with standard deviation in brackets.

† *p*-value of Fisher's exact test.

* *p*-value of _independent_ *t*-test.

### 3.2. Perioperative outcomes

**Figure 4:**
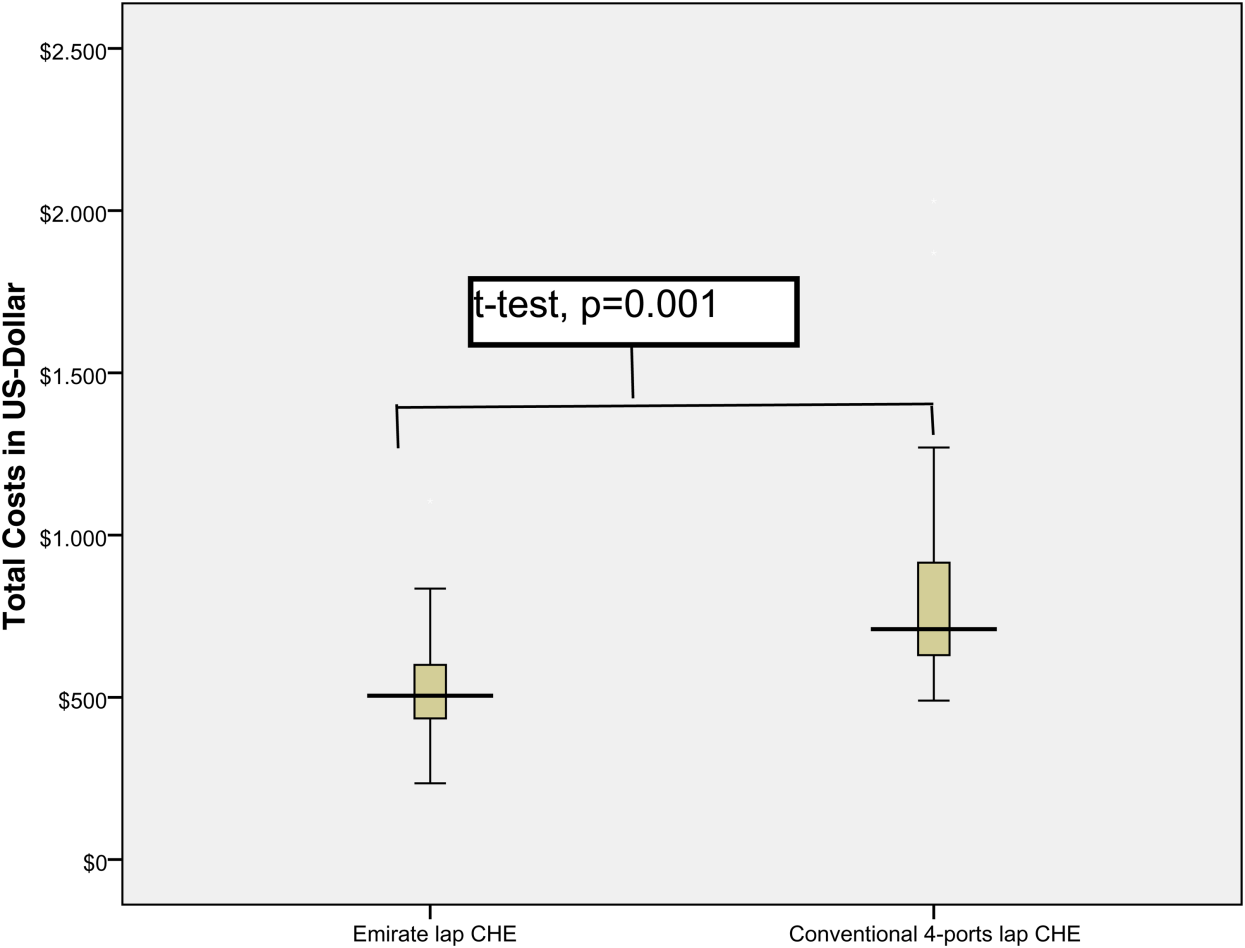
Comparison of total consumable costs and operating room utilization per minute for emirate laparoscopic cholecystectomy versus conventional four-port laparoscopic cholecystectomy.

When compared to the CLC group, the ELC group had a significantly shorter median length of operation. This difference was statistically significant (34 min in the ELC cohort and 43 min in the CLC; *t*-test, *p* = 0.0001). In addition, the overall cost of the OR was considerably lower in the ELC group ($528 in the ELC cohort as opposed to $793 in the CLC group test, *p* = 0.0001) (Figure 4). In the ELC group, the alkaline phosphatase level in serum was generally lower on the first postoperative day compared to the CLC cohort *t*-test, *p* = 0.003. This difference was statistically significant. The median length of stay in the hospital was 1.5 days across both groups (Table 2). In none of the study groups was it necessary to convert to open surgery or perform any other kind of intervention due to complications such as bile leakage, bleeding, or biliary obstruction.

**Table 2:** Post-operative data for each study group.

Variable	ELC-group (*n* = 140)	CLC-group (*n* = 140)	*p*-value
Surgery duration in minutes	34.36 (13.95)	43.36 (26.33)	**0.0001**
Length of hospital stay	1.58 (1.399)	1.54 (1.210)	0.806
OR cost per case	528.64 (139.564)	793.57 (263.39)	**0.0001**
Postop serum aspartate aminotransferase	46.15 (31.46)	43.1 (68.65)	0.806
Postop serum alanine transaminase	65.35 (71)	56.47 (114)	0.513
Postop serum alkaline phosphatase	74.98 (32.9)	94.5 (52.36)	**0.003**

ELC-group = Emirate cholecystectomy; CLC-group = conventional four-port laparoscopic cholecystectomy. Values are presented as the mean with standard deviation in brackets. *p* values less than 0.05 was deemed statistically significant are highlighted in bold.

*p*-value of Fisher's exact test.

*p*-value of _independent_ i-test.

## 4 . Discussion

In the last two decades, scarless, non-invasive cosmetic treatments have become more popular worldwide. Social media may indeed be driving the public's increased interest by offering a variety of information, from online teaching tools and physician-managed accounts to patient experiences and marketing. Promotions from celebrity accounts have been proven to have an impact on general interest. Surgeons could enhance their expertise and add innovative services by understanding more about their patients' particular interests. This is the situation with cholecystectomy, which has gradually evolved to be safer and less invasive. Furthermore, several novel laparoscopic techniques aim to improve aesthetic results while maintaining or improving therapeutic effects. Within this context, MLC, SSS, and NOTES are among the most popular and innovative minimally invasive techniques (12–14).

These techniques initially showed great promise; however, there are still some drawbacks. For example, Ma et al. (15) found that SILC had no effect on overall patient satisfaction. In addition, it has been shown that surgeons with CLC competence need additional training to perform SLIC surgery safely owing to the higher collision of surgical tools due to a lack of triangulation and the restricted number of devices that may be employed (16).

Hoyuela et al. found that SILC is linked with a statistically substantially greater long-term incisional hernia rate at the umbilical port site than CLC. According to their statistics, there was no significant advantage in terms of postoperative course, hospital stay, or aesthetic satisfaction. Ultimately, they concluded that SILC should not be used routinely (17). In a recent systematic review and meta-analysis conducted by Cirag and Schankar, it was shown that SILC had a considerably longer operational duration and more complications in comparison to CLC (18).

Despite the fact that transvaginal cholecystectomy as a NOTES procedure currently only applies to female patients, it nevertheless has several drawbacks that are equivalent to those of SILC. A further drawback of transvaginal cholecystectomy is that the majority of institutions execute transvaginal cholecystectomy as a hybrid technique, employing an abdominal trocar for the optic due to issues with instrument triangulation and the lack of flexible tools with acceptable intraperitoneal navigation. A benefit of transvaginal cholecystectomy is the decrease in postoperative pain and the need for opioids. Even for experienced laparoscopic surgeons, however, a steep learning curve using transvaginal cholecystectomy can result in a considerable lengthening of the surgery, in addition to the need for expensive specialized equipment (19). Similar issues are applicable for mini laparoscopy.

A precedent has been set for the suprapubic cholecystectomy, as demonstrated by the work of Degano et al., who described it in 1995 (20). Numerous studies comparing the suprapubic technique to the standard cholecystectomy used a variety of port sizes and numbers. Despite the use of four ports, two of which were 10 mm in the umbilical fold and 12 mm in the suprapubic region, a recent study by Taha et al. demonstrated that the aesthetic effect of the suprapubic method is superior to that of the standard cholecystectomy in a standard context, as judged not by the patient or practitioner directly involved in the therapy, but by those unrelated to the treatment process (21–23).

In the current study we analyzed 280 patients who underwent surgery because of benign gallbladder disorders at our institution (140 ELC vs. 140 CLC). The results show that the two techniques are comparable in terms of preoperative demographics, surgeon expertise, gallbladder stone size, stage of gallbladder inflammation, and preoperative bile duct diagnostics. The perioperative outcomes show that there was a statistically significant difference in the average duration of surgery; however, the ELS had an approximately 9-minute shorter operating time which is related to faster introduction of 5 mm intraumbilical optic trocar and waiving the facia closure by using pure blunt 5 mm trocar, while CLC required Hasson open technique for 10–12 mm perumbilical trocar with consecutive necessarily facia closure. Also waiving a fourth trocar as well as clipping of the artery save time when compared to CLC.

ELS also saved US$265 on average per case. Moreover, in comparison to the CLC, the laboratory parameter demonstrated much lower alkaline phosphatase serum levels. These lower AP levels, even if they have no clinical relevance, could be an expression of reduced manipulation of the gallbladder as a result of direct grasping of the infundibulum and waiving the fourth port that is usually used in the CLC to grasp the fundus with assistance.

The Emirate cholecystectomy, as a modified suprapubic laparoscopic cholecystectomy, has been designed to have a shorter running time and a less steep learning curve than SILC or NOTES. The possibility of using typical CA devices with fewer appliances makes the technique simple and cost-effective to execute. Furthermore, the entire process flow, including the patient position and order of anesthesia equipment, assistant surgeons, and the instrumenting nurse in the surgical setup, remains the same as in a conventional laparoscopic cholecystectomy. In addition to this, it is the responsibility of the academic hospital to keep laparoscopic cholecystectomy simple and easy to learn in order to ensure an adequate residency training curriculum. This is not always the case with SILS and NOTES cholecystectomies, which may be complicated and difficult to learn (24–27).

The current study is not without shortcomings. The study, which was retrospective in nature, was carried out in a single setting. In spite of this, our findings indicate that ELC is both safe and feasible in comparison to CLC, with no statistically significant differences in postoperative morbidity or mortality between the two procedures. However, this paper details the results for a sizable sample of patients who underwent ECL, and those results are similar to those for individuals who underwent CLC. Despite the fact that ELC appears to be more technically demanding than CLC at first glance because of the limited appliances, reduced number of ports, and unusual location of the optic at the suprapubic site, ELC demonstrated faster surgery times and lower costs.

## 5. Conclusions

In our study, the results of the Emirate laparoscopic cholecystectomy were similar to the results of the standard laparoscopic cholecystectomy. However, new research shows that a suprapubic cholecystectomy is less painful than a regular laparoscopic cholecystectomy. The unique technique hides the suprapubic scar with hair or clothing, which makes patients more satisfied with the cosmetic results of surgery. In order to confirm that Emirate laparoscopic cholecystectomy results in less postoperative discomfort and less visible scarring than standard laparoscopic cholecystectomy, a randomized controlled investigation is required.

## Data Availability

The datasets presented in this article are not readily available because; if needed can be provided. Requests to access the datasets should be directed to; iyad.hassan@burjeel.com.

## References

[1] Bray F Balcaen T Baro E Gandon A Ficheur G Chazard E. Increased incidence of cholecystectomy related to gallbladder disease in France: analysis of 807,307 cholecystectomy procedures over a period of seven years. J Visc Surg. (2019) 156(3):209–15. 10.1016/j.jviscsurg.2018.12.003 30573436

[2] Peery AF Crockett SD Murphy CC Lund JL Dellon ES Williams JL Burden and cost of gastrointestinal, liver, and pancreatic diseases in the United States: update 2018. Gastroenterology. (2019) 156(1):254–272.e11. 10.1053/j.gastro.2018.08.063 30315778 PMC6689327

[3] Lammert F Neubrand MW Bittner R Feussner H Greiner L Hagenmüller F S3-guidelines for diagnosis and treatment of gallstones. German society for digestive and metabolic diseases and German society for surgery of the alimentary tract. Z Gastroenterol. (2007) 45:971–1001. 10.1055/s-2007-963437 17874360

[4] Singh S Lavania S. Laparoscopic cholecystectomy: what is appropriate position of epigastric port? World J Laparoscopic Surg. (2013) 6(3):134–7. 10.5005/jp-journals-10007-1199

[5] Degano G Santarelli E Ceraudo E Iantosca F Taccaliti F Monaco M. Video-laparoscopic cholecystectomy with suprapubic approach. Tech Note Minerva Chir. (1995) 50(12):1109–14. 8725073

[6] Martins MVC Skinovsky J Coelho DE Ramos A Galvão Neto MP Rodrigues J Cholecystectomy by single trocar access (SITRACC): the first multicenter study. Surg Innov. (2009) 16(4):313–6. 10.1177/1553350609353422 20031944

[7] Gerdes B Gitei E Akkermann O Prasse-Badde J Schmidt C. Laparoscopic cholecystectomy without visible scar. Combined suprapubic and transumbilical approach: the “minden cholecystectomy”. Endoscopy. (2009) 41(Suppl 2):E49–50. 10.1055/s-0029-1214483 19294615

[8] Wood SG Dai F Dabu-Bondoc S Mikhael H Vadivelu N Duffy A Transvaginal cholecystectomy learning curve. Surg Endosc. (2015) 29(7):1837–41. 10.1007/s00464-014-3873-3 25294548

[9] Lin H Zhang J Li X Li Y Su S. Comparative outcomes of single-incision laparoscopic, mini-laparoscopic, four-port laparoscopic, three-port laparoscopic, and single-incision robotic cholecystectomy: a systematic review and network meta-analysis. Updates Surg. (2023) 75(1):41–51. 10.1007/s13304-022-01387-2 36205830

[10] Federmann GF Hessler CH. Hidden laparoscopic access (HiLA) cholecystectomy—first results. Eur Surg. (2011) 43(1):34. 10.1007/s10353-010-0575-5

[11] Ersoz F Ozcan O Sari S Bektas H Arikan S. Laparoscopic cholecystectomy on the bikini line for invisible scar. Surg Laparosc Endosc Percutan Tech. (2011) 21(1):e7–e10. 10.1097/SLE.0b013e3182064d5f 21304378

[12] Gurusamy KS Vaughan J Rossi M Davidson BR. Fewer-than-four ports versus four ports for laparoscopic cholecystectomy. Cochrane Database Syst Rev: 2014 (2):CD007109. 10.1002/14651858.CD007109.pub2 PMC10773887 24558020

[13] Thakur V Schlachta CM Jayaraman S. Minilaparoscopic versus conventional laparoscopic cholecystectomy a systematic review and meta-analysis. Surg. (2011) 253:244–58. 10.1097/SLA.0b013e318207bf52 21183848

[14] Bulian DR Knuth J Cerasani N Lange J Ströhlein MA Sauerwald A Transvaginal hybrid NOTES cholecystectomy—results of a randomized clinical trial after 6 months. Langenbecks Arch Surg. (2014) 399:717–24. 10.1007/s00423-014-1218-2 24952726

[15] Sinan H Demirbas S Ozer MT Sucullu I Akyol M. Single-incision laparoscopic cholecystectomy versus laparoscopic cholecystectomy: a prospective randomized study. Laparosc Endosc Percutaneous Tech. (2012) 22:12–6. 10.1097/SLE.0b013e3182402448 22318052

[16] Ma J Cassera MA Spaun GO Hammill CW Hansen PD Aliabadi-Wahle S. Randomized controlled trial comparing single-port laparoscopic cholecystectomy and four-port laparoscopic cholecystectomy. Surg. (2011) 254:22–7. 10.1097/SLA.0b013e3182192f89 21494123

[17] Pucher PH Sodergren MH Singh P Darzi A Parakseva P. Have we learned from lessons of the past? A systematic review of training for single incision laparoscopic surgery. Endosc. (2013) 27:1478–84. 10.1007/s00464-012-2632-6 23073688

[18] Pereira C Gururaj S. A systematic review and meta-analysis of single-incision laparoscopic cholecystectomy versus conventional four-port laparoscopic cholecystectomy. Cureus. (2022) 14(12):e32524. 10.7759/cureus.32524 36654582 PMC9840409

[19] Hoyuela C Juvany M Guillaumes S Ardid J Trias M Bachero I Long-term incisional hernia rate after single-incision laparoscopic cholecystectomy is significantly higher than that after standard three-port laparoscopy: a cohort study. Hernia. (2019) 23(6):1205–13. 10.1007/s10029-019-01969-x 31073959

[20] Bulian DR Walper S Richards DC Seefeldt CS Heiss MM. Comparative analysis of postoperative pain after transvaginal hybrid NOTES versus traditional laparoscopic cholecystectomy in obese patients. Surg Endosc. (2022) 36:4983–91. 10.1007/s00464-021-08855-7 34731301 PMC9160114

[21] Yang E Nie D Li Z. Comparison of Major clinical outcomes between transvaginal NOTES and traditional laparoscopic surgery: a systematic review and meta-analysis. Surg Res. (2019) 244:278–90. 10.1016/j.jss.2019.06.012 31302326

[22] Degano G Santarelli E Ceraudo E Iantosca F Taccaliti F Monaco M. Colecistectomia per via videolaparoscopica con approccio sovrapubico. Note di tecnica [video-laparoscopic cholecystectomy with suprapubic approach. Technical note]. Minerva Chir. (1995) 50:1109–14. 8725073

[23] Taha A Taha-Mehlitz S Sternkopf U Sorba E Enodien B Vorburger S. Suprapubic cholecystectomy improves cosmetic outcome compared to classic cholecystectomy. J Clin Med. (2022) 11(15):4579. 10.3390/jcm11154579 35956193 PMC9369808

[24] Sales LA Pinto JO Queiroz CE Castro M Dourado PH Pinheiro FA. Suprapubic laparoscopic cholecystectomy: technique and preliminary results. Bras Cir Dig. (2014) 27:22–5. 10.1590/s0102-67202014000100006 PMC4675485 24676293

[25] De la Cruz-Munoz N Koniaris L. Alternative port site selection (APSS) for improved cosmesis in laparoscopic surgery. Gastrointest Surg. (2010) 14:2004–8. 10.1007/s11605-010-1282-z 20676792

[26] Hipp J Laniewski J Gitei E Elhabash S Akkermann O Gerdes B. Operationszeit bei der suprapubisch-transumbilikalen cholezystektomie: ergebnisse einer prospektiv-randomisierten studie [operation time for suprapubic transumbilical cholecystectomy: results of a prospective randomized trial]. Die Chir. (2015) 86:866–73. 10.1007/s00104-014-2958-9 25604307

[27] Silva MV Almeida DF Alves MM Barbosa MA Vieira MW. Laparoscopic cholecystectomy with suprapubic approach. Arq Bras Cir Dig. (2013) 26(3):179–83. 10.1590/S0102-67202013000300005 24190374

